# Auxetic Liquid Crystal Elastomers: Overcoming Barriers to Scale-Up

**DOI:** 10.1021/acsapm.5c00212

**Published:** 2025-03-24

**Authors:** Stuart R. Berrow, Thomas Raistrick, Richard J. Mandle, Helen F. Gleeson

**Affiliations:** † School of Physics and Astronomy, University of Leeds, Leeds LS2 9JT, U.K.; ‡ School of Chemistry, University of Leeds, Leeds LS2 9JT, U.K.

**Keywords:** liquid crystal elastomer, auxetic, mechanical metamaterials, elastomer, network

## Abstract

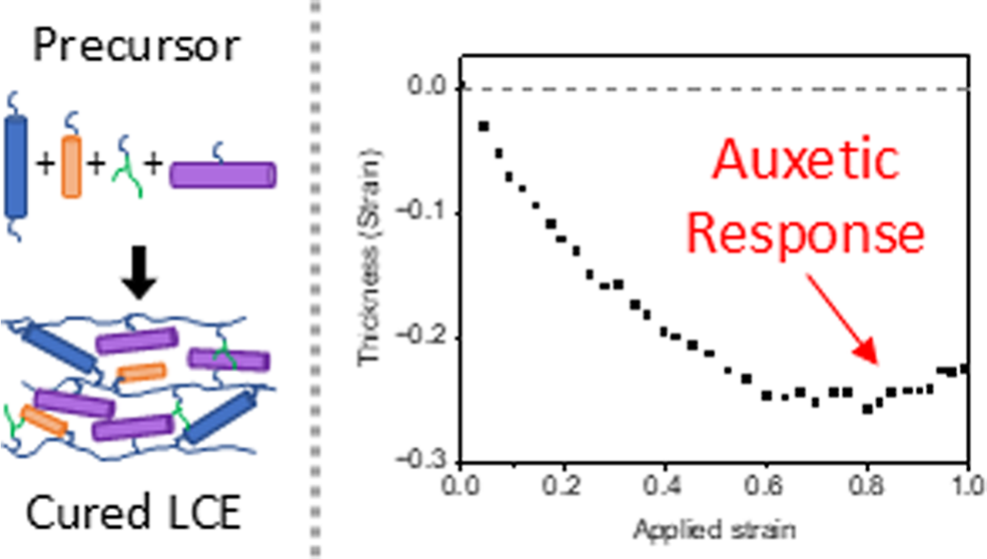

The observation of auxetic behavior (i.e., negative Poisson’s ratio) in liquid crystal elastomers (LCEs) presents an exciting opportunity to explore application areas previously inaccessible to LCEs. Since its initial discovery, research has focused on improving understanding of the underpinning physics that drives the auxetic response, the structure–property relationships that enable the response to be tuned, and LCE properties such as the refractive index. However, the auxetic LCE materials reported to date have made use of either mechanical strain during fabrication, or unreactive ‘templates’ to stabilize the nematic ordering in the precursors. The latter approach provides excellent monodomain films, but there is unavoidable anisotropic shrinkage of the LCE. Both processes previously employed create complications toward manufacturing and scale-up. In this article, we report the first example of an auxetic LCE synthesized through surface alignment without the use of a nonreactive ‘template’ and thus without the need for a washout. The LCE includes both terminally and laterally attached mesogens, presents an auxetic threshold of 76% strain, and displays a comparable dependence of auxetic behavior on its glass transition temperature as that reported in the literature. This work presents an exciting milestone in the journey toward realizing applications for auxetic LCEs.

## Introduction

Liquid crystal elastomers (LCEs) are lightly cross-linked polymers which incorporate anisotropic structural motifs known as mesogens into their structure.^1^ This leads to materials which combine the elastic properties of elastomers with the self-organization and anisotropic properties of liquid crystals. This unique combination of behaviors results in materials with interesting properties on a macroscopic level, notably actuation^1−4^ and anisotropic adhesion.^5−7^ In 2018, Mistry et al. observed a never-before-seen phenomenon for LCEs, namely auxetic behavior, more formally known as a negative Poisson’s ratio.^8^ This behavior means that the material becomes thicker rather than thinner when under strain. In the case of these LCEs, auxeticity manifests upon reaching a threshold strain when the LCE is stretched perpendicular to the nematic director. This observation marked the first synthetic, nonporous material displaying an auxetic response on a molecular level.

Auxetics are of interest to several industries, as when subject to strain they exhibit extremely desirable mechanical behaviors in comparison with traditional materials with a positive Poisson’s ratio.^9^ For example, the application of auxetics as solutions for delamination resistance, impact protection, and shock absorbance have all been suggested.^10−14^ However, the majority of auxetic materials (known as reentrant auxetics) are formed by carefully engineered porous structures, in which the pores deform or rotate under strain to yield the auxetic response.^12^ These porous structures have drawbacks that limit their suitability for some applications. For example, they are complex to fabricate, have limited scalability and are usually opaque or scatter light,^12^ which is suboptimal in cases where transparency is desirable such as in the production of impact resistant glass products.^15^ Auxetic LCEs have significant advantages: they are relatively simple to fabricate and as they are auxetic at a molecular level, the response is scalable from nanometer to macroscopic length scales.^16^ Further, because they do not have a porous structure, they are highly transparent (up to 94% transparency at 589 nm).^15^ Indeed, LCEs could readily be employed in the many application areas envisaged for traditional auxetics, in addition to some, such as those on very small scales or that require transparency, where reentrant auxetics simply cannot.^15^

The physical phenomena underpinning the auxetic response have been studied in detail. Many nematic LCEs deform uniaxially under strain perpendicular to the director; the strain causes the director to continuously rotate toward the direction of strain, and such materials are not auxetic. This is known as the semisoft elastic (SSE) response. It has been established that auxetic LCEs do not deform via the SSE response, but rather deform biaxially when subject to a strain perpendicular to the director. In such materials, a growing population of mesogens rotate out-of-plane, allowing the sample to increase in thickness beyond a material-dependent threshold, known as the auxetic threshold, driving the auxetic response.^17,18^ Further evidence of the molecular-level response has been provided through studies of optical gratings embossed into an auxetic LCE, where the nanometer-scale grating amplitude itself showed auxetic behavior with a positive effect on the diffraction efficiency as the grating was strained.^16^ Attention has recently turned to systematic studies of the effect of variations to the chemical composition on the auxetic response in the LCEs, elucidating valuable information regarding the design of future auxetic LCEs.^19^

In all but one of LCEs displaying an auxetic response reported so far, an unreactive, low molecular weight mesogenic material (6OCB in Figure S1) has been employed in the precursor mixture used for LCE synthesis.^8,15−20^ The role of this unreactive mesogen is to enhance the stability of the nematic phase of the precursor mixture, the state in which the polymerization is initiated, enabling an LCE with nematic ordering to be produced. This unreactive component is then removed from the final LCE by washing with a solvent. The only example of an auxetic LCE fabricated without an unreactive component was reported by Liu and Zhao, where mechanical alignment is employed to impart the nematic ordering, with samples being strained by 100% of their original length to yield monodomains.^21^

Regardless of the method employed thus far in the literature, the fabrication of these auxetic LCEs presents issues when considering scale up. In the case of the Liu and Zhao material, mechanical stretching of a partially cured sample is impractical on large scales, particularly if a reel-to-reel process is desired. In the case of the other auxetic LCEs, the ‘washout’ step presents challenges for scale-up. Practically, the biggest challenge associated with the washout step is the anisotropic shrinkage of the LCE upon removal of the 6OCB. While in a few cases, this can be utilized to yield interesting materials,^16^ for the most part such deswelling adds complexity to the production process and limits the attainable sample size. Further issues relating to the deswelling step include sustainability concerns arising from the use of large quantities of solvent to wash the samples, and the associated cost of energy and resources in managing the waste generated.

It is therefore highly desirable to simplify the production of auxetic LCEs, eliminating the need for a mechanical straining or ‘washout’' step. In this work, we report the first example of an auxetic LCE synthesized using surface alignment without the use of an unreactive component. This methodology thereby bypasses the need for a either mechanical strain or a ‘washout’ step during fabrication, simplifying production, and is an important step toward commercialization.

## Experimental Section

### Materials and Methods

All materials were used as purchased without further purification and were obtained from one of the following suppliers: Sigma-Aldrich (Gillingham, UK), Fisher Scientific (Loughborough, UK), Apollo Scientific (Stockport, UK), Ambeed (Arlington Heights, IL, USA), Fluorochem (Glossop, UK), Tokyo Chemical Industry UK (Oxford, UK).

### Monomer Synthesis

**Scheme 1:**
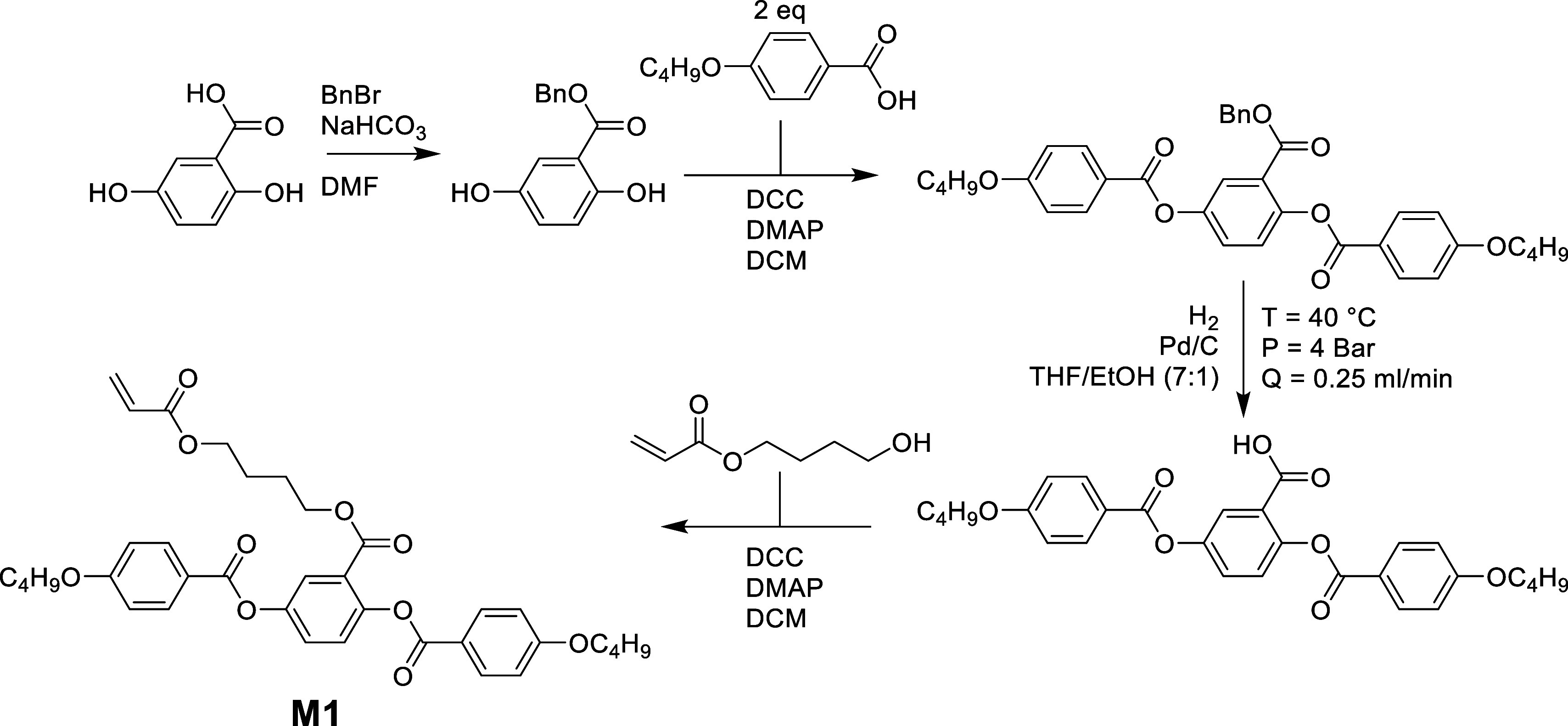
The Synthetic Procedure Employed in the Synthesis of Monomer M1

The synthesis of the laterally attached monofunctional monomer (4-acryloylbutyl)-2,5-di(4-butyloxybenzyloxy)benzoate (M1) was adapted from previous literature.^22^Scheme 1 shows the full synthetic route employed, and detailed experimental procedures can be found in the Supporting Information.

### Elastomer Mold Fabrication

The LCEs were synthesized in bespoke alignment molds, which were made in accordance with the previous literature.^17,19,20^ A glass microscope slide (7.5 cm × 2.5 cm × 1 mm) and a Melinex ST725 substrate (7 cm × 2.5 cm × 250 μm) (DuPont Teijin Films, Redcar, UK) were spin-coated on one surface with an aqueous 0.5 wt % poly(vinyl alcohol) (PVA) solution, which was uniaxially rubbed with a bespoke rubbing machine after drying the substrates at 50 °C for 15 min. These two substrates were then adhered, via Melinex 401 spacers (7.5 cm × 0.2 cm × 100 μm) (DuPont Teijin Films, Redcar, UK) and UVS-91 adhesive (Edmund Optics, York, UK), so that the PVA-rubbed surfaces were the inner surfaces of the constructed cell, and the rubbing directions yielded antiparallel planar alignment along the width of the mold. The adhesive was then cured by irradiation under 350 nm (2.5 Wcm^–2^) at 50 °C for 10 min, to yield the constructed LCE mold with a gap thickness of ∼100 μm.

### Liquid Crystal Elastomer Synthesis

In a typical procedure, 2-Methyl-1,4-phenylene bis(4-((6-(acryloyloxy)hexyloxy)benzoate (RM82) (3.5 mol %), 6-(4′-Cyanobiphenyl-4-yloxy)hexyl acrylate (A6OCB) (24.4 mol %), and (4-acryloylbutyl)-2,5-di(4-butyloxybenzyloxy)benzoate (M1) (49.6 mol %) were heated to 120 °C with stirring until a homogeneous isotropic phase was obtained. The mixture was cooled to 50 °C, followed by the addition of 2-ethylhexyl acrylate (EHA) (21 mol %) and Methyl benzoylformate (MBF) (1.5 mol %), and stirred for 5 min, again ensuring a homogeneous, completely isotropic material was obtained. The mixture was then filled into a mold at 50 °C via pipette, while still in the isotropic state, before being cooled into the nematic phase at room temperature and allowed to stand for 20 min, ensuring that uniform, monodomain alignment, promoted by the rubbed substrates in the mold, was achieved. The samples were then cured under 350 nm (2.5 Wcm^–2^) irradiation for 2 h, to yield a fully cured sample. After curing, the samples were removed from the molds (using a small amount of isopropanol if necessary to aid delamination from the substrates). If required, the samples were allowed to stand under ambient conditions overnight to allow for the loss of any isopropanol used to aid delamination.

### Material Analysis

Information regarding the analytical procedures employed in this work can be found in Supporting Information. Briefly these include: NMR spectroscopy, mass spectrometry, differential scanning calorimetry, polarized-light optical microscopy, small and wide-angle X-ray scattering and mechanical analysis.

## Results and Discussion

### Monomer Selection and Precursor Mixture Behavior

In all previous studies of auxetic LCEs, it has been found that for an LCE to display an auxetic response, the LCE must exhibit a nematic phase.^8,23^ The aim of this work was to produce an LCE capable of displaying an auxetic response without the need for the unreactive mesogen that imparted sufficient nematic phase stability. Typically, as much as ∼55 mol % 4′-hexyloxy-(1,1′-biphenyl)-4-carbonitrile (6OCB) was included in the precursor mixture. We required a monomer that would have comparable phase behavior to that of 6OCB, providing a nematic precursor mixture with no unreactive mesogenic content.

A promising candidate, (4-acryloylbutyl)-2,5-di(4-butyloxybenzyloxy)benzoate, in this work known as M1, was identified from the literature.^22^ This monomer has previously been reported to exhibit a nematic phase from 72 to 98 °C,^22^ which compares reasonably well with the nematic phase range of 60–77 °C observed for 6OCB. This suggested that M1 could be capable of achieving a similar or better phase stabilization to that imparted by 6OCB. Additionally, the laterally attached nature of the acrylate group relative to the mesogenic long axis promotes the formation of nematic phases, inhibiting smectic phase formation, and thus adding further potential to the use of this monomer.^1,24^

**Figure 1:**
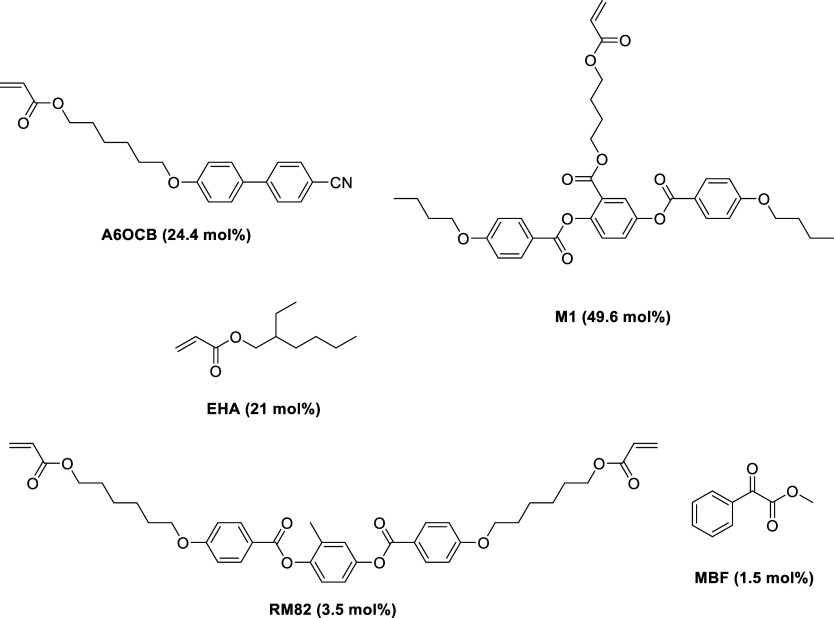
The precursor mixture used to produce the auxetic LCEs. The nematic to isotropic phase transition temperature of this mixture is 47 °C.

**Figure 2:**
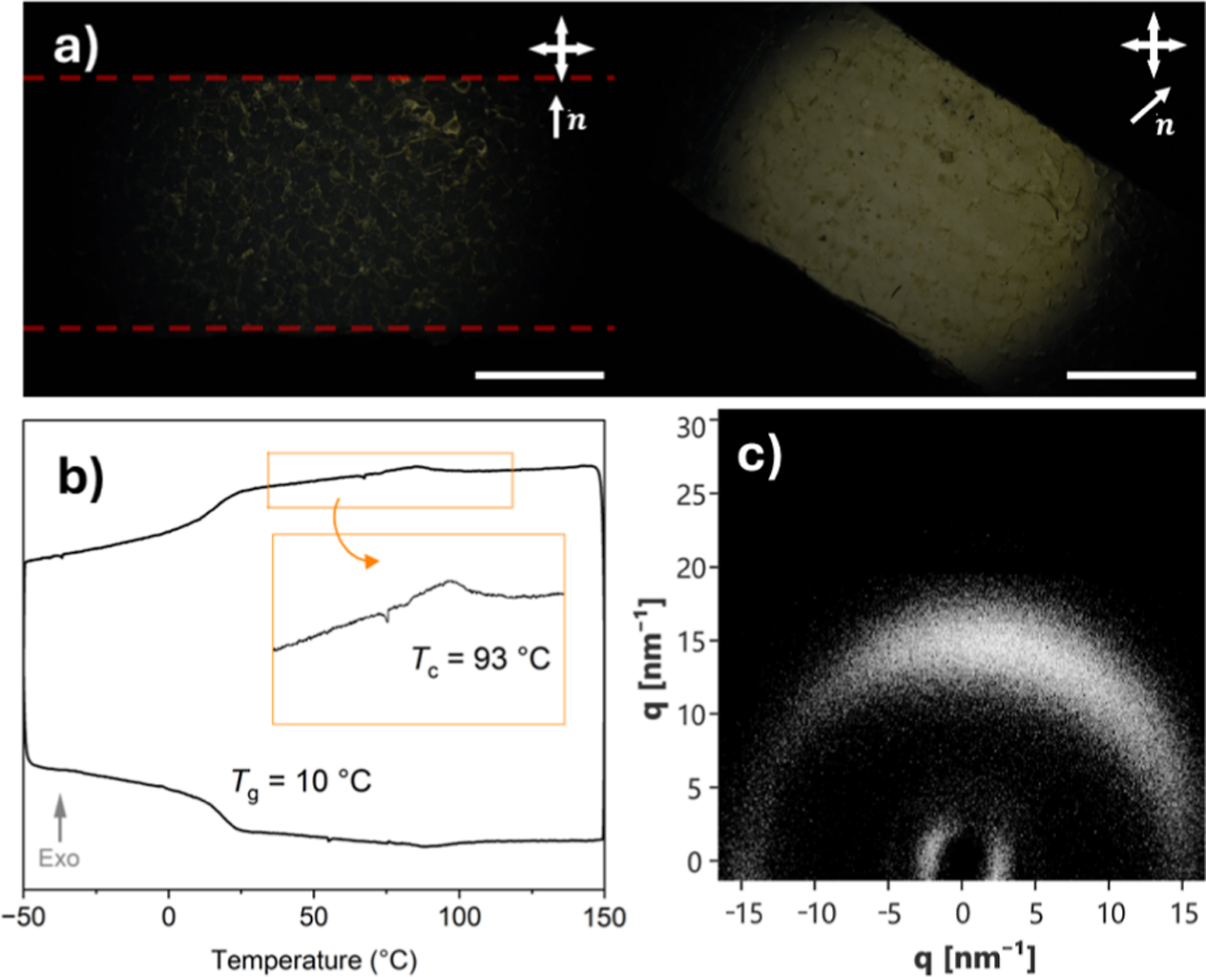
(a) POM images showing macroscopic planar alignment within the LCEs with scale bars denoting 1 mm, (b) an example DSC thermogram for the LCE recorded using heating/cooling rates of 5 °C/min, and (c) two-dimensional X-ray scattering data confirming the nematic phase of the LCE.

The precursor mixture used is displayed in Figure 1, and takes inspiration from the mixtures used in previous auxetic LCEs (Figure S1). In this work, the 54.6 mol % of 6OCB has been replaced by 49.6 mol % M1, and an additional 5 mol % EHA. The increase in the quantity of EHA by 5 mol % ensures that the LCE produced from the mixture retains a glass transition temperature (*T*_g_) below room temperature (Figure 2b). When the mixture was examined by differential scanning calorimetry (DSC) and polarized light optical microscopy (POM) (Supporting Information, Figures S2 and S3 respectively), a nematic phase was observed, stable to below room temperature with a clearing temperature of 47 °C. Thus, the precursor mixture was deemed suitable for the production of nematic LCEs at room temperature.^19^

### LCE Production and Phase Behavior

Upon irradiation with UV light, the samples cured to yield free-standing LCE films. The final LCEs are isolated as samples of approximately 7.5 cm × 2 cm × 100 μm, with only minimal variations in size resulting from an essentially negligible contraction in sample dimensions upon curing, as is typical for acrylate polymerizations.^25,26^ This is contrary to the large deswelling reported for all previous surface aligned auxetic LCEs, which often shrink in excess of 30% in the direction perpendicular to the director.^16^ The fact that the LCE reported here does not deswell makes the targeting of a given material size facile, and thus is advantageous for potential commercialization.

The central advantage of eliminating the deswelling step is that the auxetic LCEs could be fabricated in a more varied manner, widening their scope. For example, the LCEs could be fabricated in situ, such as between glass plates. Furthermore, we envision that this development offers a simplified route to scale-up of the material through other polymer fabrication processes, for example, extrusion or 3D printing. It is also possible that in a reel-to-reel process, the desired alignment could be imparted on the materials as they are deposited onto a production line, for example using aligned laminating surfaces, and the materials cured by UV irradiation. Such processes have been used in the production of chiral nematic liquid crystal polymers by BASF in the early 2000s, for anticounterfeiting applications. Furthermore, a reel-to-reel process is used by Kent Displays for the production of their Boogie Boards, a process in which the use of surface alignment is also important. While efforts would be required to optimize the conditions used during these processes (such as cure time) for auxetic LCEs, we envision that the ability to produce these samples without a washout step would allow films or fibers to be made in a continuous manner.

As with all the previous work on auxetic LCEs, nematic LCEs with macroscopic planar alignment were targeted.^8,15,17−20^ This alignment was confirmed via POM, as the samples show excellent extinction when the director is aligned with either the polarizer or analyzer and color inversion upon rotation about 45° (see Figure 2a). The phase behavior of the LCE was studied via DSC and X-ray scattering. The LCE shows a glass transition temperature (*T*_g_) at 10 °C, and a broad phase transition around 93 °C (Figure 2b). X-ray scattering (Figures 2c and S4) confirms the presence of a nematic phase at 25 °C, via the presence of oriented WAXS signals. It is of note that while there are also small angle (SAXS) peaks in the data, these are diffuse and relatively low in intensity, differing from the strong SAXS peaks that are indicative of smectic ordering.^19^ The order parameter (⟨P2⟩) was calculated from the WAXS data, and is found to be 0.60 at room temperature, typical of well-aligned, monodomain side-chain nematic LCEs.^8,15,18−20^ Based on these observations, we attribute the transition at 93 °C to be a clearing transition, where the LCE undergoes a phase transition into the isotropic phase, and therefore denote this transition as the clearing temperature (*T*_c_).

The auxetic LCEs reported to date have all exhibited a lack of any apparent phase transitions other than a *T*_g_ prior to thermal degradation.^27^ However, it should be noted that the nematic to isotropic transition in LCEs can be broadened, meaning that it would be difficult to observe via DSC or POM.^27−31^ A study by Raistrick et al. suggested that a high clearing temperature is an important requirement for LCEs displaying an auxetic response, as it allows the biaxial deformation required to produce auxeticity to dominate over other potential deformation mechanisms.^18^ The observation that this LCE has a phase transition at 93 °C may allow us to elucidate further information as to how high a clearing temperature is needed in order to allow this biaxiality to dominate.

### Auxetic Behavior

**Figure 3:**
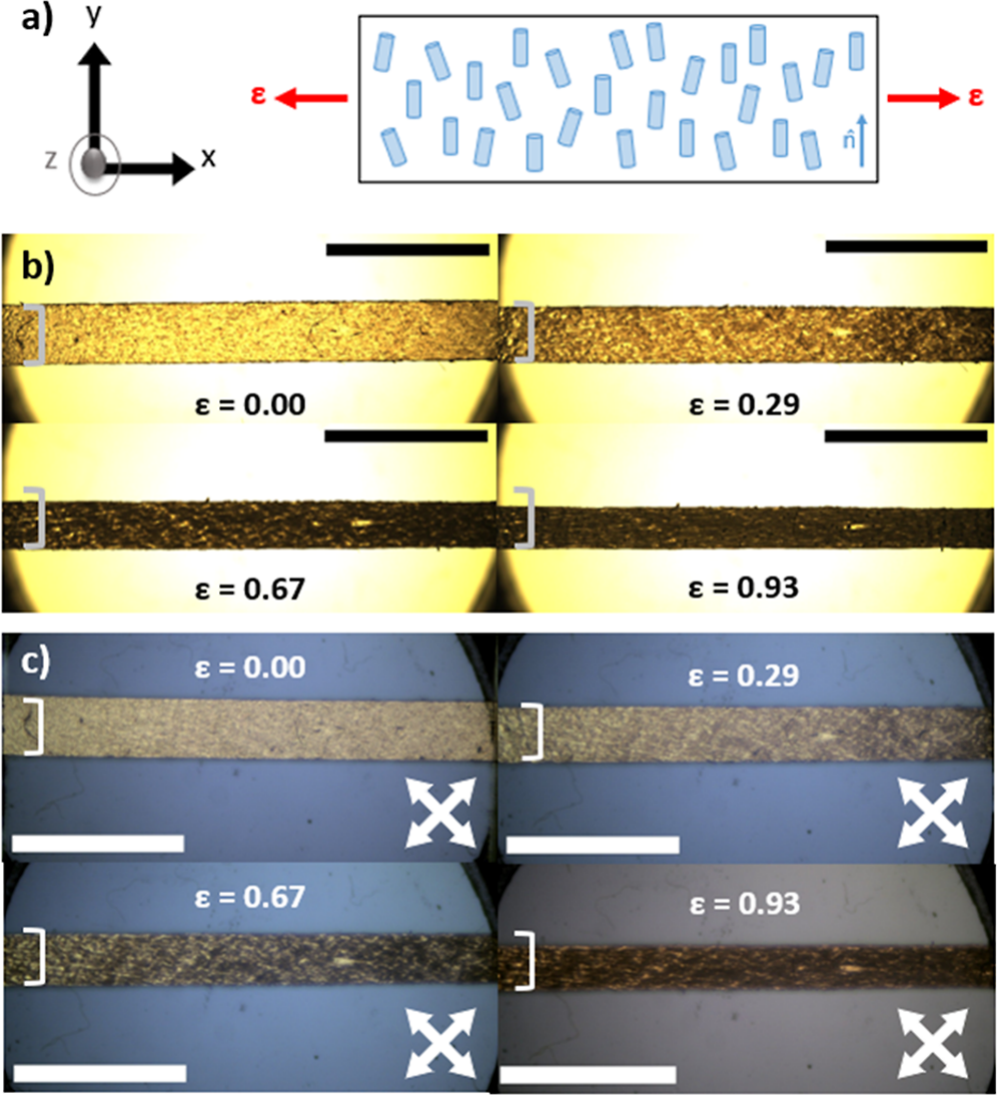
(a) A schematic representation of the mechanical analysis undertaken, displaying the nematic director (n̂) and the direction of applied strain (ε), and (b) some example images for the LCE captured during the strain experiments. (c) Example images for the LCE captured during the strain experiment under cross-polarized light conditions. The strains (ε) applied in the *x*-direction are noted for each image. A black or white horizonal scale bar represents 5 mm in (b) and (c) respectively and in (c) the directions of crossed polarizers are shown by crossed arrows. Square brackets (]) are added to aid visualization of the width changes occurring upon the application of strain; a dramatic reduction in width is seen for strains of 0.67 and 0.93 which are above the auxetic threshold of this material, coinciding with an increase in the sample thickness.

The presence of an auxetic response in these LCEs was investigated by monitoring the macroscopic shape change observed as the LCE is subject to strain.^8,17,19,20^ In brief, the sample (of dimensions 20 mm × 2 mm × 100 μm) is loaded into two actuators, and actuators separated to a distance that suitably removes any slack from the samples. The unstrained sample thickness is measured accurately using a precision micrometer. The samples are then subject to strain steps of 0.5 mm (the minimum strain step allowed by the apparatus), in intervals of 10 min, until sample failure. During this straining process, the samples are imaged to examine the macroscopic deformation. The images captured are taken from above the sample (*z*-direction, as displayed schematically in Figure 3a), and the local strains are measured in the x- and *y*-directions by tracking multiple particles on the image with a resolution of ±3 μm.^8^ By also assuming conservation of volume, which has been shown to be valid for auxetic LCEs, the strains in both transverse axes are then calculated (eq S1).^8^ The sample displays planar alignment (as indicated in Figure 3a), and strain is applied perpendicular to the nematic director. This alignment results in anisotropic behavior, meaning the samples behave differently in the y and *z* axes, and the auxetic response is observed in the film thickness (the *z* axis in Figure 3a). Although this is not directly observed in the collected images, the auxetic response can be observed indirectly as a dramatic decrease in the width of the sample in the recorded images (Figure 3b,c) as the thickness increases and the volume is conserved. This method also allows for the collection of stress–strain data, a representative example of which is given in Figure S5.

Figure 3b shows the dramatic reduction in width (*y*-axis) of the samples upon application of strain in the *x*-axis. One notable difference seen in these images when considered in conjunction with observations previously reported for auxetic LCEs is that a change in the opacity of the sample occurs as it is strained. In this case, the sample is observed to become more opaque as strain is applied, particularly around the auxetic threshold. In the unstrained state, the sample has a transmission of 81% at 589 nm when corrected for Fresnel losses, but upon the application of strain this falls as low as 21% (at 589 nm). Previous auxetic LCEs had transparency of >90% in the unstrained state and showed minimal changes in transparency upon strain.^8^ We hypothesize that the further change in opacity observed on strain is the result of a difference in the relaxation dynamics allowed in these LCEs relative to other auxetic LCEs, perhaps due to the combination of mesogen orientations (i.e., side-on and end-on as opposed to exclusively end-on) that is not present in other auxetic samples. The increased opacity may limit the scope of application of these particular LCEs to ones where good transparency is not a prerequisite, but future optimization of material compositions could overcome this issue.

The variation in opacity also complicates the interpretation of observations made under crossed polarizers (Figure 3c). Previously, the appearance of a dark state has been taken as a signature of the biaxial deformation and the resulting auxetic response.^17,18^ While similar behavior is observed in these samples, it is difficult to decipher if this is the result of mesogen reorientation and thus biaxiality, a change in opacity, or indeed a combination of both.

**Figure 4:**
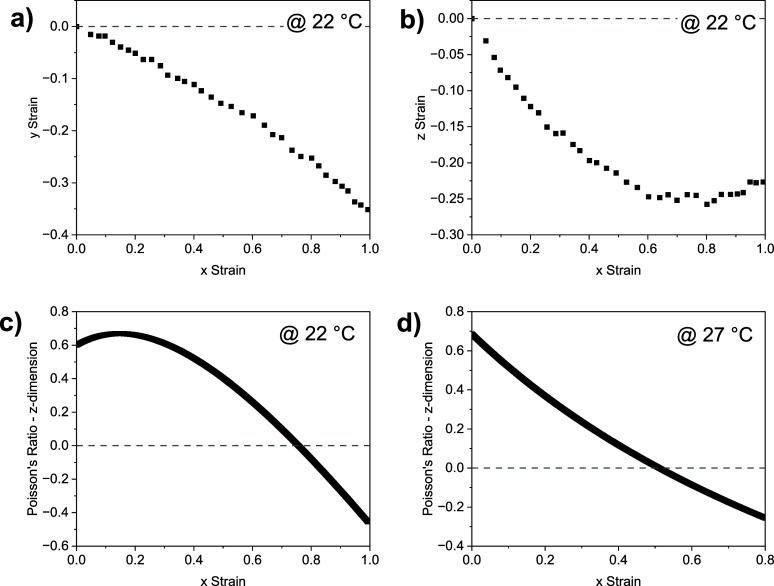
The effect of an applied x-strain on (a) the strain observed in the *y*-axis (width) of the sample at 22 °C; (b) the strain calculated in the *z*-axis (thickness) of the sample when strained at 22 °C; (c) the instantaneous Poisson’s ratio calculated in the z-dimension at 22 °C; (d) the Poisson’s ratio calculated in the z-dimension when the sample is strained at 27 °C.

The impact of an applied *x*-strain on the measured strain of the sample in both the *y* and *z* axes (as defined in Figure 3a) is displayed in Figure 4a,b. As described above, throughout the entire experiment, the sample is observed to reduce in size in the *y*-dimension. In the *z*-dimension, upon application of strain, the sample is observed to reduce in size initially, before reaching a threshold applied *x*-strain at which point the sample begins to get thicker. This behavior is consistent with that observed for previous auxetic LCEs.^8,17,19^

The auxetic threshold strain, at which point the auxeticity is observed i.e. the Poisson’s ratio becomes negative, in the case of this LCE is 0.76 (±0.05) at room temperature. Previous studies have shown that the auxetic threshold in auxetic LCEs is influenced by several factors, including the proximity to the *T*_g_ of the sample.^19^ In general, samples with higher *T*_g_ show a higher auxetic threshold when strained at room temperature.^19^ The observation of an auxetic threshold of 0.76 for this LCE, which has a *T*_g_ of 10 °C, is consistent with the auxetic thresholds reported previously for LCEs with *T*_g_ in the region of 9–12 °C which have auxetic thresholds of 0.65–0.81 respectively.^19^ Furthermore, it has previously been observed that when examined at the same reduced temperature relative to *T*_g_, the auxetic threshold of LCEs converge, in the region of 0.52–0.60.^19^ In the previous literature, the reduced temperature reported is 1.06× *T*_g_ (in K), which for the LCE reported in this work, is equivalent to 27 °C. When this LCE is strained at 27 °C (Figure 4d), an auxetic response is still observed, with an auxetic threshold of 0.51. We suggest that this evidence further supports the observations that *T*_g_ has an influence on the auxetic behavior of these LCEs.

## Conclusions

This work details the first example of an auxetic liquid crystal elastomer synthesized using surface alignment without the need for an unreactive component, and thus a washout step, during the synthesis. This is achieved using a side-on liquid crystal monomer, which imparts the same nematic phase stabilization on the LCE precursor mixture as the previously used unreactive materials. The result is a precursor mixture that has a stable nematic phase at room temperature, thereby facilitating facile LCE synthesis at ambient temperatures. When subject to strain the LCE initially displays a positive Poisson’s ratio, and thus gets thinner, before reaching a threshold value at which point the Poisson’s ratio becomes negative and the sample thickens, consistent with the behavior of previous auxetic LCEs. The auxetic threshold strain for this LCE at room temperature is 0.76 (i.e., 76% strain). When the *T*_g_ (10 °C) of the sample is considered, and the LCE strained at a reduced temperature of 1.06 relative to *T*_g_, the auxetic threshold reduces to 0.51, consistent with the convergence of auxetic thresholds previously reported.

Not only are these findings exciting from a point of view of scientific interest, but they could also have a profound impact on the potential commercialization and application of auxetic LCEs. All previous auxetic LCEs either require the removal of unreactive material from the LCE films post cure, which results in anisotropic deswelling often of the order of >30% perpendicular to the nematic director, or a mechanical straining step to impart alignment. By eliminating the need for such processes, sample fabrication is streamlined. In the case of the previous LCEs that require a washout, the shrinkage observed upon deswelling makes targeting a specific sample size complex. The method employed in this work leads to changes in sample dimensions that are negligible, and thus targeting a given sample size for a desired application/product is easier. This is in addition to the prevention of the generation of large quantities of waste material that the deswelling processes would have caused, thereby improving the overall sustainability of the fabrication processes. Finally, as already noted, the removal of the wash-out step allows the LCE films to be formed more easily using bulk approaches such as roll-to-roll methodologies or in situ, for example between glass plates. Neither of the previous methodologies would facilitate such possibilities. This work therefore represents a significant milestone in the utilization of auxetic LCEs.

## Data Availability

The data underlying this study are openly available in Data set associated with “Auxetic Liquid Crystal Elastomers: Overcoming Barriers to Scale-Up”, available at 10.5518/1614.

## Supporting Information Available

Detailed experimental information and experimental procedure, Structural confirmation of the synthesized monomer and intermediates, and phase transition analysis of the LCE precursor mixture via differential scanning calorimetry and polarized light optical microscopy (PDF)

## Abbreviations

6OCB: 4′-Hexyloxy-(1,1′-biphenyl)-4-carbonitrile
A6OCB: 6-(4′-Cyanobiphenyl-4-yloxy)hexyl acrylate
DSC: differential scanning calorimetry
EHA: 2-ethylhexyl acrylate
LCE: liquid crystal elastomer
M1: (4-acryloylbutyl)-2,5-di(4-butyloxybenzyloxy)benzoate
MBF: methyl benzoylformate
POM: Polarized light optical microscopy
PVA: Poly(vinyl alcohol)
RM82: 2-Methyl-1,4-phenylene bis(4-((6-(acryloyloxy)hexyloxy)benzoate
SAXS: Small-angle X-ray scattering
*T*_g_: glass transition temperature
WAXS: Wide-angle X-ray scattering.
