# Supplementary material for: Auxetic Liquid Crystal Elastomers: Overcoming Barriers to Scale-Up
Source: ACS Appl Polym Mater. 2025 Mar 24;7(7):4517–24. doi: 10.1021/acsapm.5c00212 (PMC11997952; doi:10.1021/acsapm.5c00212)
Supplement: Supplementary file 1 — ap5c00212_si_001.pdf [file ap5c00212_si_001.pdf]

Supporting Information

Auxetic Liquid Crystal Elastomers Overcoming Barriers to Scale-Up

Stuart R. Berrow[1*], Thomas Raistrick[1], Richard J. Mandle[1,2], and Helen F. Gleeson[1]

[1]School of Physics and Astronomy, University Of Leeds, Leeds, UK, LS2 9JT

[2]School of Chemistry, University of Leeds, Leeds, UK, LS2 9JT

*Correspondence: S.R.Berrow@leeds.ac.uk

Supporting Information available: Experimental information, synthetic procedures for the synthesis of (4-acryloylbutyl)-2,5-di(4-butyloxybenzyloxy)benzoate (M1), generalised precursor mixture employed in previous auxetic LCEs, phase behaviour analysis for the LCE precursor mixture, X-ray scattering patterns collected as a function of temperature for the cured LCEs, and a stress-strain curve for the LCEs.

Table Of Contents

Experimental Information S2
Flash Chromatography S2
Structural Analysis S2
Thermal Analysis S2
Optical Microscopy S2
X-Ray Scattering Measurements S3
Mechanical Analysis S3
Synthetic Procedures S5
LCE Precursor Mixture Employed in Previous Work S8
Precursor Mixture Phase Transitions S9
Variable Temperature X-Ray Scattering S11
Stress-Strain Data S12
References S13

Experimental Information

Flash Chromatography

Flash chromatography was performed on a Combiflash NextGen 300+ system (Teledyne Isco) using silica as a stationary phase, an appropriate mobile phase as specified in the experimental procedure, and detection in the 200-800 nm wavelength range.

Structural Analysis

Nuclear magnetic resonance (NMR) spectra were recorded using a Bruker AVANCE III (400 MHz) NMR spectrometer (Bruker UK Ltd., Coventry, UK) at 298 K and referenced to TMS. NMR spectra were viewed and analysed using MNova NMR software.

Accurate Mass spectra were acquired on a Bruker Impact II QqTOF spectrometer equipped with a VIPHESI source using electrospray ionisation. Samples were introduced using an HTC PAL autosampler and Bruker Elute Pump. HPLC columns were heated to 40 °C unless otherwise stated. Samples passed through a Bruker Diode array UV-detector before entering the mass spectrometer. Calibration was performed by infusion of 5mM sodium formate solution at the end of each acquisition. Samples were submitted as solutions in acetonitrile at 10 µg/mL concentration, and data were collected in positive mode.

Thermal Analysis

Differential scanning calorimetry (DSC) measurements were performed using a TA Instruments Q2000 DSC instrument (TA Instruments, Wilmslow UK), equipped with a RCS90 Refrigerated cooling system (TA Instruments, Wilmslow UK). The instrument was calibrated against an Indium standard, and data were processed using TA Instruments Universal Analysis Software. Samples were analysed under a nitrogen atmosphere, in hermetically sealed aluminium TZero crucibles (TA Instruments, Wilmslow, UK) and subjected to three analysis cycles.

For the analysis of the synthesised monomers each cycle consisted of: a heating phase from 0–140 °C at a heating rate of 10 °C/min, an isothermal phase at 140 °C for 2 minutes, a cooling phase from 140–0 °C at 10 °C/min, and an isothermal phase at 0 °C for 2 minutes. Melting points are reported as peak values on the first heating cycle, and clearing temperatures are reported as onset temperatures on the first cooling cycle.

For the analysis of the uncured LCE mixtures, each cycle consisted of: a heating phase from 0–100 °C at a heating rate of 10 °C/min, an isothermal phase at 100 °C for 2 minutes, a cooling phase from 100–0 °C at 10 °C/min, and an isothermal phase at 0 °C for 2 minutes. Clearing temperatures are reported as onset values.

For LCE analysis, each cycle consisted of: a heating phase from -50–150 °C at a heating rate of 10 °C/min, and isothermal phase at 150 °C for 2 minutes, a cooling phase from 150 – -50 °C at 10 °C/min, and an isothermal phase at -50 °C for 2 minutes. The glass transition temperatures of the polymers were recorded as the onset value, on the heating phase of the second cycle.

Optical Microscopy

Polarised light optical microscopy (POM) was performed using a Leica DM2700P polarised light microscope (Leica Microsystems (UK) Ltd., Milton Keynes, UK), equipped with 10x and 50x magnification, and a rotatable stage. For LCE samples, the films were mounted on a glass slide, and analysed under ambient conditions, using 10x magnification. For phase

identification of the liquid crystal mixtures, a Mettler Toledo FP82HT Hot Stage (Mettler-Toledo Ltd., Leicester, UK), controlled by a Mettler Toledo FP90 central processor (Mettler-Toledo Ltd., Leicester, UK) was used to control the temperature of the sample with a relative accuracy of 0.1 °C. In this case, the sample was mounted between a glass microscope slide and a glass cover slip, and samples were analysed using 50x magnification. Images were recorded using a Nikon D3500 Digital Camera (Nikon UK Ltd., Surbiton, UK), using DigiCamControl software.

X-Ray Scattering Measurements

2D Small angle (SAXS) and wide angle (WAXS) X-ray scattering experiments were performed on an Anton Paar SAXSpoint 5.0 system (K-α Cu source, λ=1.5418 Å) with a Dectris EIGER2 R 1M (1028 pixel x 1062 pixel array). Measurements were performed at room temperature on 100 µm (nominal thickness) LCE samples averaging 5 frames with exposures times of 60 s. A 2 mm beam size was used and the measurements were run using a beam stop-less set-up. A background scan was performed for both the SAXS and WAXS detector position which was subtracted from the measurements to minimise contributions from intrinsic background scattering and the Mylar protective film in front of the detector. 2D data reduction was performed by radially integrating the 2D patterns whilst masking the central contribution related to the non-scattered X-ray beam.

The scalar nematic order parameter, S, was calculated from the 2D WAXS pattern using the Kratky method[1] shown in **equation S1**:

$$I(\chi) = f_0 + \frac{1}{2}f_2\cos^2(\chi) + \frac{3}{8}f_4\cos^4(\chi) + \frac{5}{16}f_6\cos^6(\chi) + \frac{35}{128}f_8\cos^8(\chi) + \frac{63}{256}f_{10}\cos^{10}(\chi) \ldots, \quad \textbf{(S1)}$$

Where I is intensity, χ is the azimuthal angle orthogonal with respect to the nematic director and f_n are constants determined by fitting **equation S1**. The f_n constants are used to determine $\langle\cos^2(\beta)\rangle$ through the following equation:

$$\langle\cos^2(\beta)\rangle = \frac{\sum_{n=0}^{\infty}\frac{f_{2n}}{2n+3}}{\sum_{n=0}^{\infty}\frac{f_{2n}}{2n+1}}, \quad \textbf{(S2)}$$

where β is the angle between the axis of a molecule and the nematic director. Thus $S = \langle P_2\rangle$ can be determined through the following equation:

$$S = \frac{1}{2}\left(3\langle\cos^2(\beta)\rangle - 1\right). \quad \textbf{(S3)}$$

In calculating S, the baseline was removed using a zero-offset method.

Mechanical Analysis

Mechanical measurements were conducted using bespoke equipment designed and manufactured in-house, full specifications for which can be found in previous work.[2,3] This apparatus consists of two actuators and a load cell, enclosed within a temperature-controlled environment, and is equipped with optics that enable images of the sample to be recorded both via optical microscopy and polarising optical microscopy simultaneously. In this work, samples of 20 mm x 2 mm were analysed at room temperature. The initial gap between the actuators was 16.5 mm, and the samples were subject to strain steps of 0.5 mm at 10-minute intervals, until the sample failed. The samples were strained perpendicular to the initial nematic director, as displayed in **Figure 3a)** in the article text. Changes in sample

width and length were monitored by particle tracking in the recorded images. Strain in sample thickness (ε_z) can be calculated based on conservation of volume, via **equation S2**, where a sample of initial volume $l_0 \times w_0 \times t_0$ maintains constant volume when strained to dimensions $l \times w \times t$:

$$\varepsilon_z = \frac{t}{t_0} - 1 = \frac{l_0}{l} \times \frac{w_0}{w} - 1 \quad \textbf{(S2)}$$

Synthetic Procedures

Synthesis of Benzyl-2,5-dihydroxybenzoate

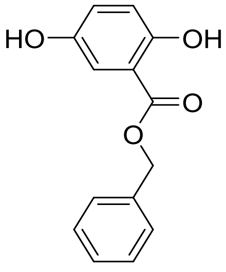

To DMF (100 mL) was added 2,5-dihydroxybenzoic acid (6.16 g, 40 mmol) and sodium hydrogen carbonate (9.9 g, 118 mmol), and the resulting suspension heated to 70 °C and stirred for 1 hour. To the mixture was added benzyl bromide (6.84 g, 40 mmol) and the suspension stirred at 70 °C overnight. The reaction mixture was cooled to room temperature, and diluted with water (200 mL). The resulting aqueous medium was extracted with 1:1 hexane:ethyl acetate (4x100 mL), and the combined organic phase washed with water (2x100 mL) and dried over magnesium sulphate. The solvent wash removed under reduced pressure, and the resulting crude product was purified over silica gel using diethyl ether as the eluent, to yield as a pale orange solid of mass 7.75 g (79 % yield).

^{1}H NMR (400 MHz, $CDCl_3$) δ 10.29 (s, 1H, -O**H**), 7.36 – 7.22 (m, 5H, Ar**H**), 7.26 – 7.17 (m, 1H, Ar**H**), 6.90 (dd, *J* = 8.9, 3.1 Hz, 1H, Ar**H**), 6.77 (d, *J* = 8.9, 1H, Ar**H**), 5.25 (s, 2H, -C**H**$_2$-), 4.84 (s (broad), 1H, -O**H**).

^{13}C NMR (101 MHz, $CDCl_3$) δ 169.58 (**C**=O), 155.82, 147.83 (Ar**C**-O), 135.17, 128.76, 128.64, 128.41, 128.32, 124.27, 118.55, 114.87, 112.23 (Ar**C**), 67.15 (**C**-O).

Synthesis of Benzyl 2,5-di(4-butoxybenzoyloxy)benzoate

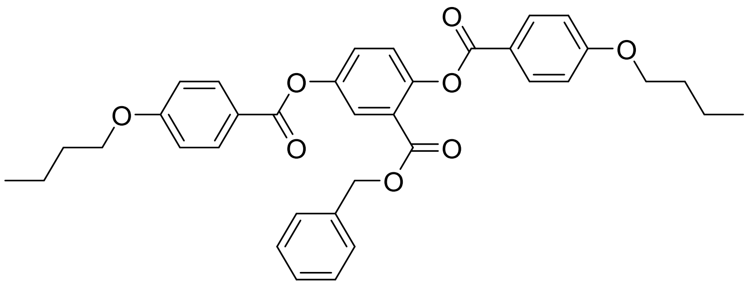

To DCM (400 mL) was added Benzyl-2,5-dihydroxybenzoate (6 g, 24.6 mmol), 4-butoxybenzoic acid (10.5 g, 54 mmol), N,N'-dicyclohexylcarbodiimide (11.14 g, 54 mmol) and 4-dimethylaminopyridine (0.66 g, 5.4 mmol), and the solution left to stir at room temperature overnight. The resulting suspension was filtered under gravity, and the filtrate washed with water (150 mL), 5% aqueous acetic acid solution (150 mL) and brine (150 mL), and the organic layer dried over anhydrous sodium sulphate. The solvent was removed under reduced pressure and the resulting solid recrystallized from ethanol to yield the product as a colourless solid of mass 10.79 g (74% yield).

^{1}H NMR (400 MHz, $CDCl_3$) δ 8.10 – 8.03 (m, 2H, Ar**H**), 8.03 – 7.95 (m, 2H, Ar**H**), 7.83 (d, *J* = 2.9 Hz, 1H, Ar**H**), 7.39 (dd, *J* = 8.8, 2.9 Hz, 1H, Ar**H**), 7.22 – 7.12 (m, 2H, Ar**H**), 6.94 – 6.87 (m, 2H, Ar**H**), 6.87 – 6.80 (m, 2H, Ar**H**), 5.12 (s, 2H,-OC**H**$_2$-), 3.98 (td, *J* = 6.5, 1.1 Hz, 4H, ,-

OCH_2-), 1.75 (dtd, *J* = 14.3, 6.5, 3.7 Hz, 2H, -C**H**$_2$-), 1.53 – 1.38 (m, 6H, -C**H**$_2$-), 0.93 (td, *J* = 7.4, 4.7 Hz, 6H, -C**H**$_3$).

^{13}C NMR (101 MHz, $CDCl_3$) δ 164.97, 164.60, 163.86 (**C**=O), 163.77, 163.52, 148.32, 148.27 (Ar**C**-O), 135.25, 132.43, 132.40, 128.52, 128.43, 128.21, 127.34, 125.06, 124.63, 121.32, 121.00, 114.39, 114.29 (Ar**C**), 68.06, 67.99, 67.25 (**C**-O), 31.16, 31.14, 19.23, 19.21 (-**C**H$_2$-), 13.84 (-**C**H$_3$).

Synthesis of 2,5-di(4-butoxybenzoyloxy)benzoic Acid

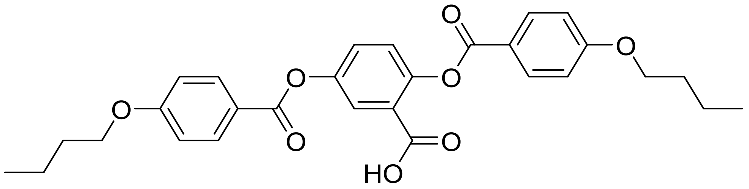

A solution of 0.05M Benzyl 2,5-di(4-butoxybenzoyloxy)benzoate in 7:1 tetrahydrofuran:ethanol was passed through an H-cube, operating at 40 °C, at a rate of 0.25 mL/min. The H-cube was loaded with a 10% Pd/C catalyst, and hydrogen gas of pressure 5 bar was pumped into the reactor. The solution was fed through the reactor cyclically, and conversion monitored via TLC. When the reaction had completed, the solvent was removed under reduced pressure, to yield the product as a colourless solid (99% yield).

^{1}H NMR (400 MHz, $CDCl_3$) δ 8.10 – 8.03 (m, 2H, Ar**H**), 7.83 (d, *J* = 2.9 Hz, 1H, Ar**H**), 7.41 (dd, *J* = 8.8, 2.9 Hz, 1H, Ar**H**), 7.22 (d, 1H, Ar**H**), 6.94 – 6.87 (m, 2H, Ar**H**), 6.87 – 6.80 (m, 2H, Ar**H**), 3.98 (td, *J* = 6.5, 1.1 Hz, 4H, ,-OC**H**$_2$-), 1.75 (dtd, *J* = 14.3, 6.5, 3.7 Hz, 2H, -C**H**$_2$-), 1.53 – 1.38 (m, 6H, -C**H**$_2$-), 0.93 (td, *J* = 7.4, 4.7 Hz, 6H, -C**H**$_3$).

^{13}C NMR (101 MHz, $CDCl_3$) δ 168.86, 164.96, 164.55 (**C**=O), 148.76, 148.30, (Ar**C**-O), 132.48, 132.42, 132.32, 128.53, 128.15, 125.51, 125.24, 123.48, 123.17, 121.28, 120.93, 118.63, 114.41, 114.33 (Ar**C**), 68.07, 68.00 (-**C**-O-), 31.18, 30.34, 19.22 (-**C**H$_2$-), 13.85 (-**C**H$_3$).

Synthesis of (4-acryloylbutyl)-2,5-di(4-butyloxybenzyloxy)benzoate (M1)

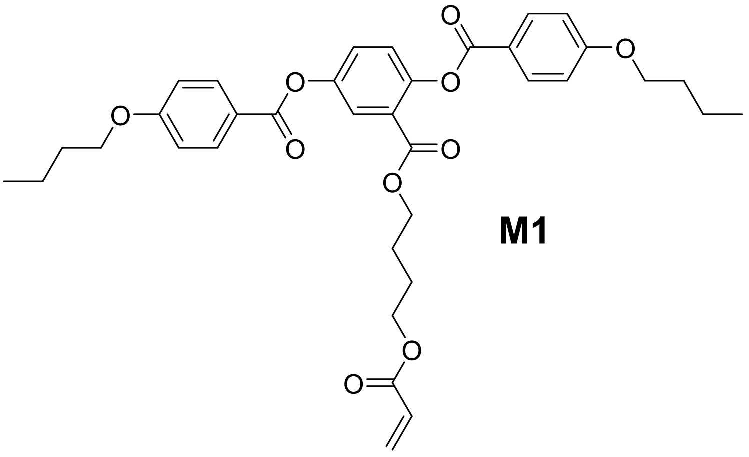

M1

To DCM (180 mL) was added 2,5-di(4-butoxybenzoyloxy)benzoic Acid (5.04g, 10 mmol), 4-hydroxybutyl acrylate (1.87 g, 13 mmol), N,N'-dicyclohexylcarbodiimide (2.68 g, 13 mmol)

and 4-dimethylaminopyridine (0.16 g, 1.3 mmol). The resulting mixture was stirred at room temperature overnight. The resulting suspension was filtered under gravity, and the filtrate washed with water (100 mL), 5% aqueous acetic acid solution (100 mL) and brine (100 mL), and the organic layer dried over anhydrous sodium sulphate. The solvent was removed under reduced pressure, and the resulting solid recrystallized from ethanol, to yield the product as a colourless solid of mass 3.46 g (55% yield).

^{1}H NMR (400 MHz, $CDCl_3$) δ 8.22 – 8.11 (m, 4H, Ar**H**), 7.91 (d, *J* = 2.9 Hz, 1H, Ar**H**), 7.48 (dd, *J* = 8.8, 2.9 Hz, 1H, Ar**H**), 7.32 – 7.23 (m, 1H, Ar**H**), 7.05 – 6.91 (m, 4H, Ar**H**), 6.39 (dd, *J* = 17.3, 1.5 Hz, 1H, CH$\mathbf{H_{trans}}$), 6.10 (dd, *J* = 17.3, 10.5 Hz, 1H, =C**H**), 5.82 (dd, *J* = 10.4, 1.5 Hz, 1H, CH$\mathbf{H_{cis}}$), 4.23 (t, *J* = 6.1 Hz, 2H, -OC$\mathbf{H}_2$-), 4.14 – 3.99 (m, 6H, -OC$\mathbf{H}_2$-), 1.90 – 1.78 (m, 4H, -C$\mathbf{H}_2$-), 1.73 – 1.48 (m, 4H, -C$\mathbf{H}_2$-), 1.23 (d, *J* = 6.1 Hz, 4H, -C$\mathbf{H}_2$-), 1.02 (td, *J* = 7.4, 0.8 Hz, 6H, -C$\mathbf{H}_3$).

^{13}C NMR (101 MHz, $CDCl_3$) δ 166.12, 164.93, 164.60, 164.06, 163.78, 163.68 (**C**=O), 148.36, 148.12, 132.44, 132.41 (Ar**C**), 130.67, 128.43 (**C=C**), 127.23, 125.05, 125.00, 124.80, 121.35, 121.02, 114.41 (Ar**C**), 114.38, 68.07, 68.04, 64.90, 63.91 (-**C**-O-), 31.14, 25.15, 19.21 (-**C**H_2-), 13.84 (-**C**H_3).

LCE Precursor Mixture Employed in Previous Work

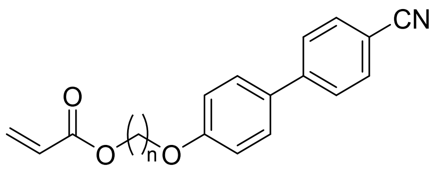

AnOCB

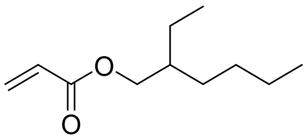

EHA

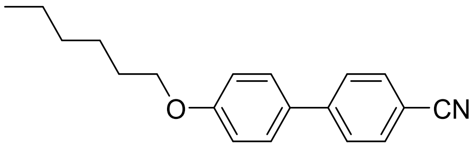

6OCB

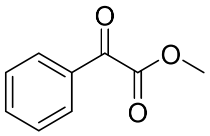

MBF

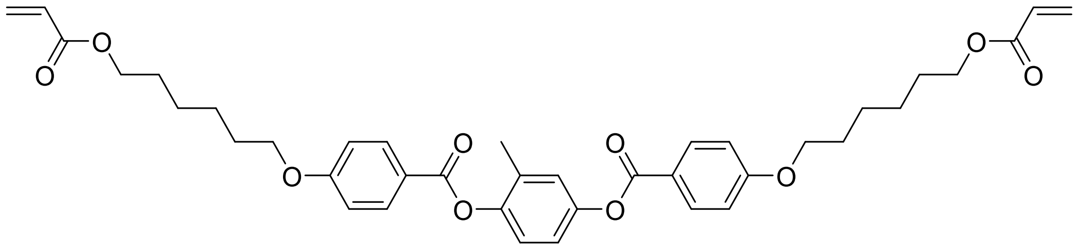

RM82

Figure S1 *– The components employed in the previous auxetic LCEs reported in the literature, that employ unreactive material to impart phase stability.*[2–7]

Precursor Mixture Phase Transitions

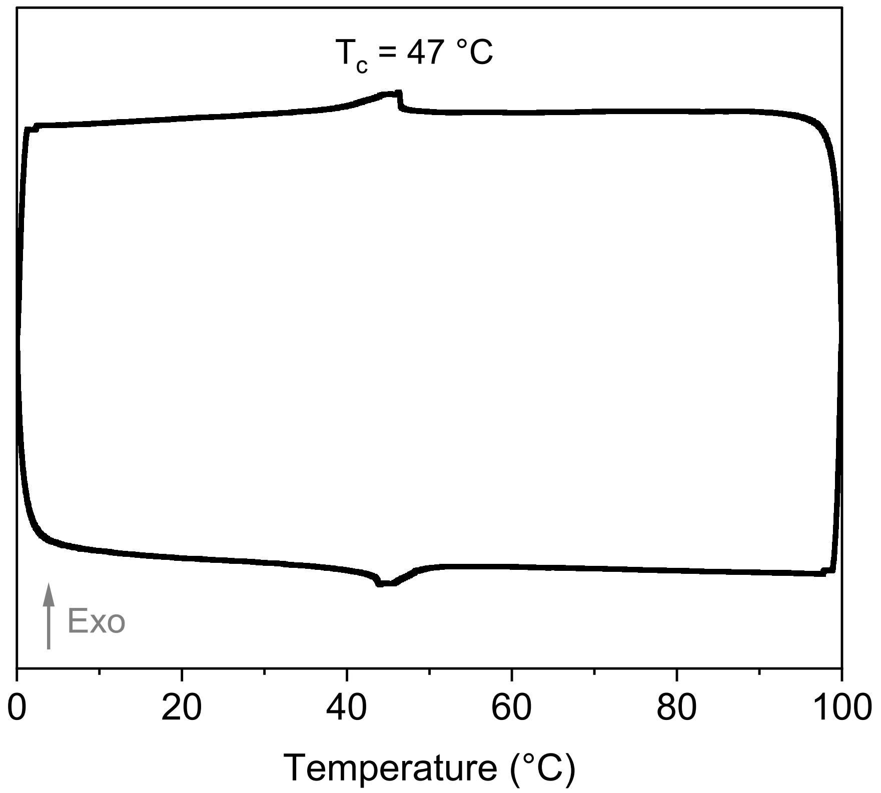

Figure S2 *– DSC thermogram for the LCE precursor mixture reported in this work.*

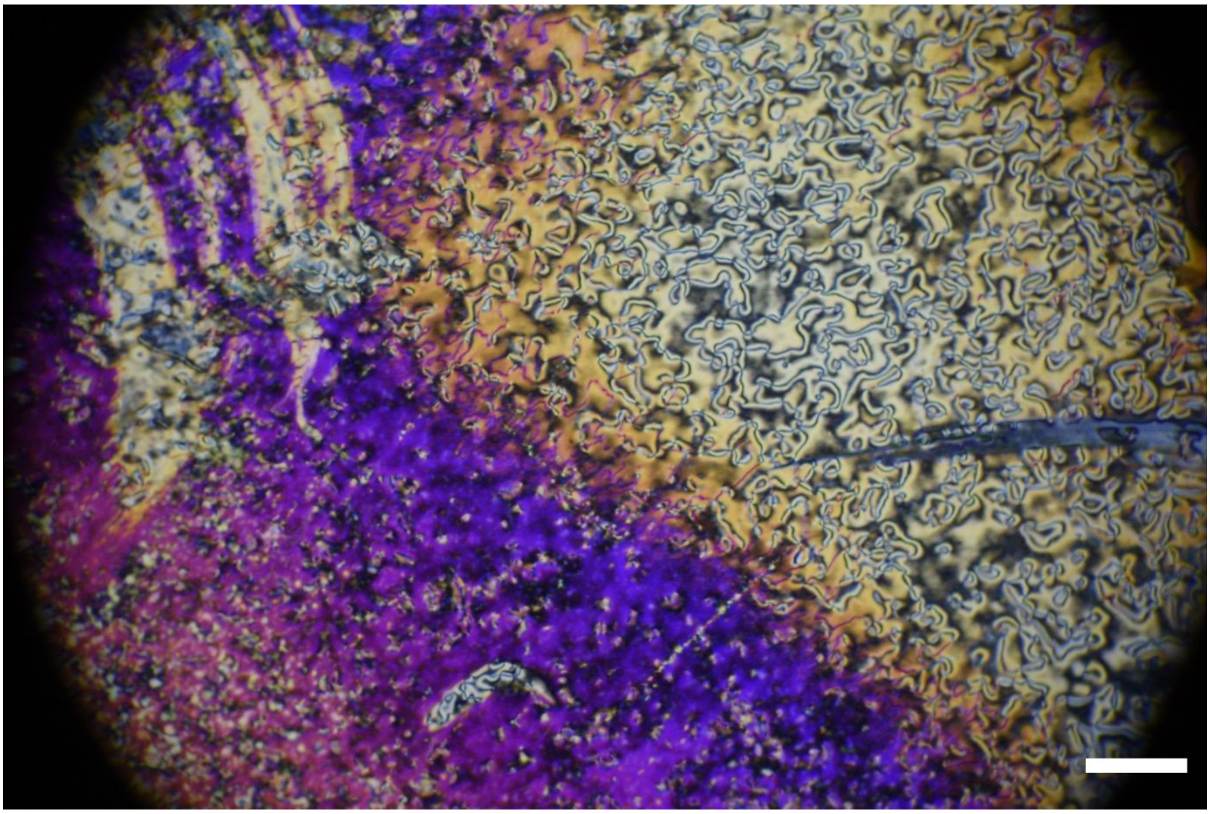

***Figure S3** – The nematic phase of the LCE precursor mixture, displaying a characteristic Schlieren texture when viewed under crossed-polarisers. (Image obtained from sample affixed between two untreated glass substrates at a temperature of 40 °C. The scale bar represents 100 μm).*

Variable Temperature X-Ray Scattering

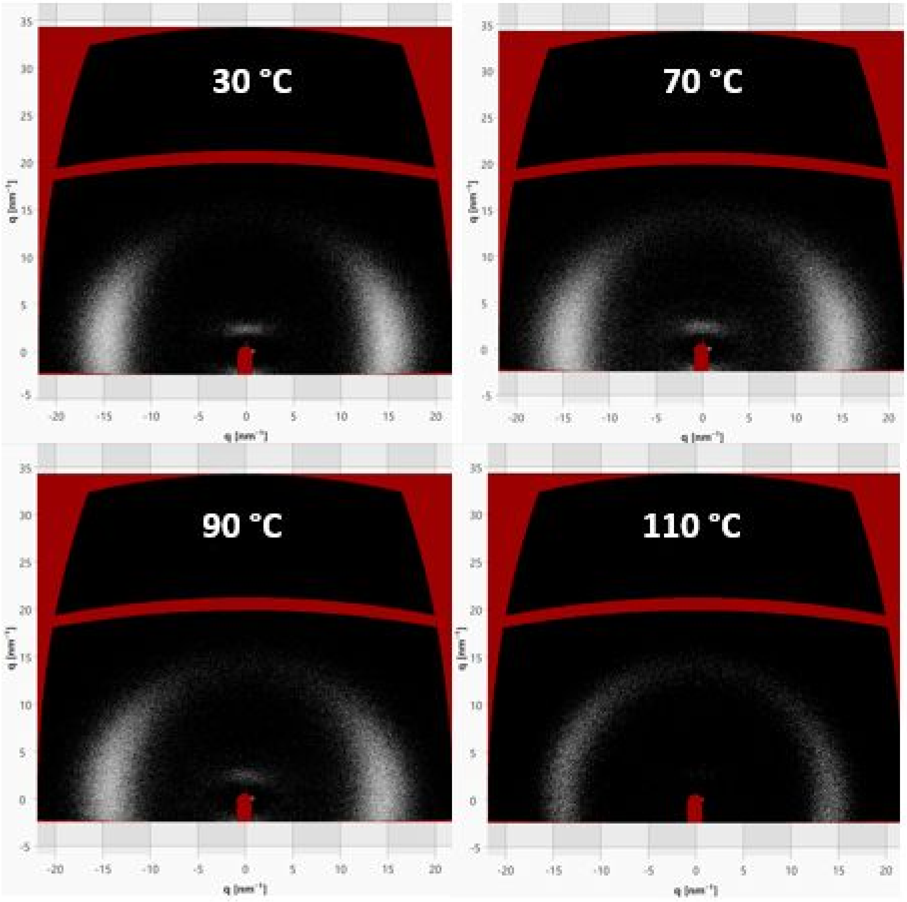

Figure S4 *– X-ray scattering patterns obtained as a function of temperature for the cured LCE. The pattern obtained at 110 °C shows a broadening of scattering pattern, indicative of a reduction in order, in this case attributed to a nematic to isotropic phase transition.*

Stress-Strain Data

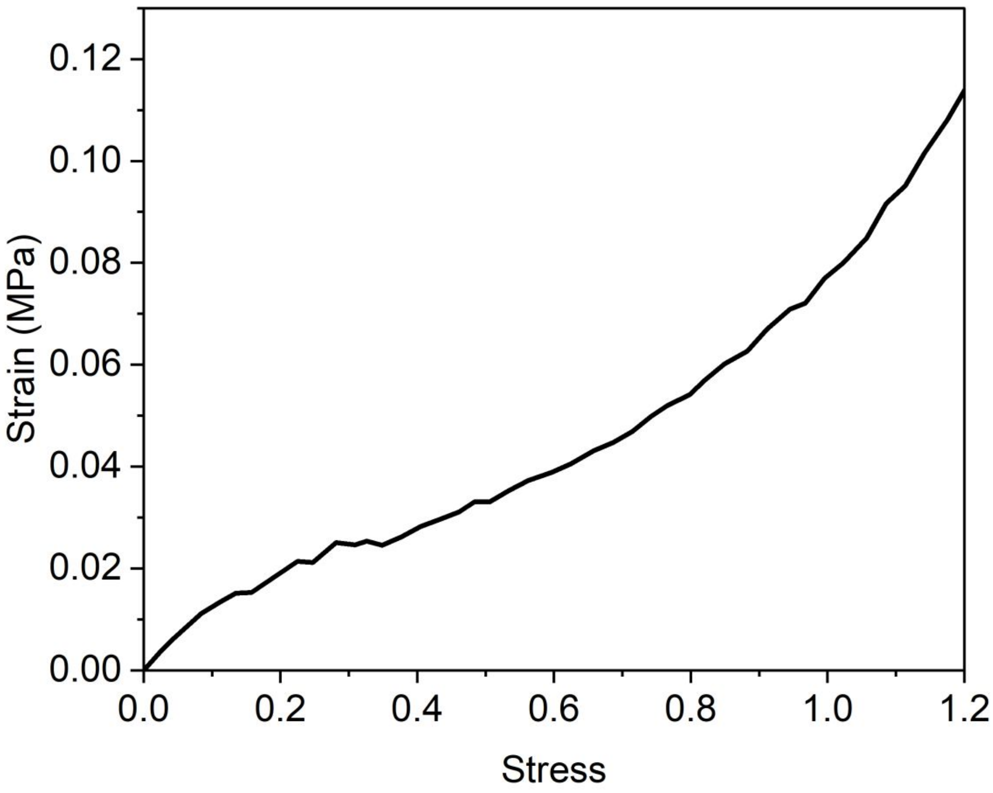

***Figure S5** – An example stress-strain curve for the LCEs, recorded upon the application of strain in 0.5 mm increments at 10-minute intervals, in accordance with the procedure detailed on pages S3 and S4. The x-axis represents engineering strain.*

References

(1) Sims, M. T.; Abbott, L. C.; Richardson, R. M.; Goodby, J. W.; Moore, J. N. Considerations in the Determination of Orientational Order Parameters from X-Ray Scattering Experiments. *Liq Cryst* **2019**, *46* (1), 11–24. https://doi.org/10.1080/02678292.2018.1455227.

(2) Mistry, D.; Connell, S. D.; Mickthwaite, S. L.; Morgan, P. B.; Clamp, J. H.; Gleeson, H. F. Coincident Molecular Auxeticity and Negative Order Parameter in a Liquid Crystal Elastomer. *Nat Commun* **2018**, *9* (1), 5095. https://doi.org/10.1038/s41467-018-07587-y.

(3) Raistrick, T.; Zhang, Z.; Mistry, D.; Mattsson, J.; Gleeson, H. F. Understanding the Physics of the Auxetic Response in a Liquid Crystal Elastomer. *Phys Rev Res* **2021**, *3* (2), 023191. https://doi.org/10.1103/PhysRevResearch.3.023191.

(4) Moorhouse, T.; Raistrick, T. Sub-Micron Diffractive Optical Elements Facilitated by Intrinsic Deswelling of Auxetic Liquid Crystal Elastomers. *Adv Opt Mater* **2024**, *12* (24), 2400866. https://doi.org/10.1002/adom.202400866.

(5) Berrow, S. R.; Raistrick, T.; Mandle, R. J.; Gleeson, H. F. Structure–Property Relationships in Auxetic Liquid Crystal Elastomers—The Effect of Spacer Length. *Polymers (Basel)* **2024**, *16* (14), 1957. https://doi.org/10.3390/polym16141957.

(6) Cooper, E. J.; Reynolds, M.; Raistrick, T.; Berrow, S. R.; Jull, E. I. L.; Reshetnyak, V.; Mistry, D.; Gleeson, H. F. Controlling the Optical Properties of Transparent Auxetic Liquid Crystal Elastomers. *Macromolecules* **2024**, *57*, 2030–2038. https://doi.org/10.1021/acs.macromol.3c02226.

(7) Wang, Z.; Raistrick, T.; Street, A.; Reynolds, M.; Liu, Y.; Gleeson, H. F. Direct Observation of Biaxial Nematic Order in Auxetic Liquid Crystal Elastomers. *Materials* **2023**, *16* (1), 393. https://doi.org/10.3390/ma16010393.
